# Transient Suppression of Dopamine Transporter Palmitoylation by Methamphetamine: Implications for Transport Regulation

**DOI:** 10.1096/fj.202501472R

**Published:** 2025-10-30

**Authors:** Moriah J. Hovde, Danielle E. Bolland, Corey D. Kleinsasser, Madhur Shetty, Aaron C. Blackwell, Mikhail Y. Golovko, Svetlana A. Golovko, Christopher R. Brown, James D. Foster, Roxanne A. Vaughan

**Affiliations:** ^1^ Department of Biomedical Sciences University of North Dakota, School of Medicine and Health Sciences Grand Forks North Dakota USA

**Keywords:** acyl protein thioesterase, amphetamine, cocaine, palmitoyl acyl transferase, post translational modification, protein kinase C, protein palmitoyl thioesterase, psychostimulant drugs, striatum

## Abstract

The dopamine transporter (DAT) exerts temporal and spatial control over dopaminergic neurotransmission through reuptake of extracellular dopamine (DA). The functional capacity of DAT is under the control of signaling inputs and post‐translational modifications that confer acute presynaptic regulation of reuptake in response to physiological needs, and dysregulation of these processes may contribute to DA imbalances in mood disorders and drug addiction. A key modification of DAT is palmitoylation, a lipid adduction that enhances transport velocity, is suppressed by protein kinase C, and opposes protein kinase C‐mediated down‐regulation. Here we now show in rat striatum and heterologous cells that transporter palmitoylation is also linked to methamphetamine (METH), undergoing rapid and transient reductions in response to the drug. The time course and other characteristics of palmitoylation reduction parallel those of METH‐induced transport down‐regulation, and a palmitoylation‐deficient DAT mutant shows enhanced down‐regulation to METH, supporting a mechanistic link between reduction of the modification and reduced reuptake activity. Recovery rates differed, however, with palmitoylation returning to starting levels more rapidly than reuptake, indicating that down‐regulation mechanisms remain engaged with transporters that have undergone repalmitoylation. These results support palmitoylation as a rapid response mechanism that modulates the entry of DAT into METH‐induced down‐regulation states and suggest a broader role for the modification in control of reuptake in additional physiological and pathophysiological conditions.

## Graphical abstract

This study examines the effects of METH on dopamine transporter (DAT) palmitoylation, a modification that enhances transport velocity and opposes PKC‐induced down‐regulation. In rats injected with METH, DAT palmitoylation was suppressed within minutes followed by a gradual return to control levels. METH‐induced transport down‐regulation occurred within a similar time frame, but recovery was significantly slower. This suggests that palmitoylation opposes DAT entry into METH‐induced down‐regulation states associated with psychomotor stimulation and reward, but may not strongly impact transport recovery.

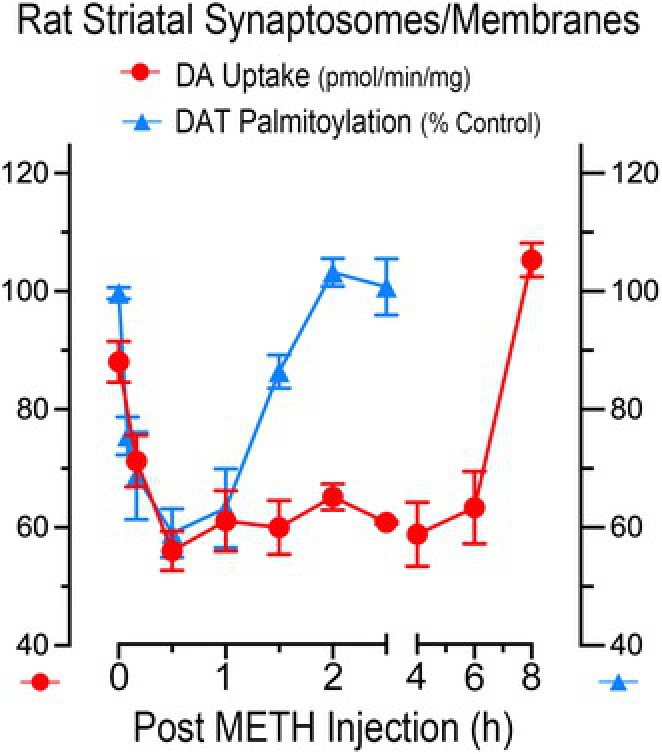

**Abbreviations**
ABE: acyl biotinyl exchange
AMEM: alpha minimal essential medium
AMPH: amphetamine
APT: acyl protein thioesterase
BCA: bicinchoninic acid
BIM 1: bisindolylmaleimide 1
DA: dopamine
EDTA: ethylenediaminetetraacetic acid
HEPES: 4‐(2‐hydroxyethyl)‐1‐piperazineethanesulfonic acid
HPDP biotin: sulfhydryl‐reactive *N*‐(6‐(biotinamido)hexyl)‐3′‐(2′‐pyridyldithio)‐propionamide
KRH: Krebs‐Ringers‐HEPES buffer
LLC‐PK_1_
: Lily Lab Cells Porcine Kidney
METH: methamphetamine
NA: hydroxylamine
NEM: N‐ethylmaleimide
PAT: palmitoyl acyltransferase
PKC: protein kinase C
PMSF: phenylmethanesulfonyl fluoride
PPT: palmitoyl protein thioesterase
RIPA: radioimmunoprecipitation assay
SP: sucrose phosphate buffer
STX: syntaxin 1A

## 1 Introduction

The dopamine transporter (DAT) is expressed on plasma membranes of axons and terminals of midbrain dopaminergic neurons that project to the basal ganglia and cortical areas of the brain [1]. DAT plays a key role in dopamine (DA) neurotransmission in these regions through reuptake of extracellular transmitter, and dysregulation of DAT function may result in dopaminergic imbalances in mood, psychiatric, and movement disorders [2]. Many abused drugs, including transport blockers such as cocaine and transported substrates such as amphetamine (AMPH) and methamphetamine (METH), act at DAT to inhibit DA reuptake, elevating transmitter to supraphysiological levels that underlie psychomotor stimulation and addiction [3, 4].

In addition to their pharmacological actions, AMPH and METH affect DAT physiologically by inducing down‐regulation of reuptake through kinetic and endocytotic processes that persist after the drug has been cleared, which further increases the magnitude and duration of transport reductions and contributes to addictive potential [5, 6, 7]. Under normal conditions, DAT activity and surface expression are regulated by multiple signaling systems that synergistically function to coordinate transmitter clearance with physiological needs [7]. A major pathway linked to transport reduction and endocytosis is protein kinase C (PKC), and the similarities between AMPH‐ and METH‐induced down‐regulation events and those of PKC have drawn attention to post‐translational modifications in psychostimulant mechanisms. PKC stimulates phosphorylation of DAT N‐terminal domain residues that mediate reduced reuptake [8, 9, 10, 11, 12]. Phosphorylation of this domain is also stimulated by AMPH and METH in a PKC‐dependent manner, implicating the kinase as a key element in psychostimulant substrate actions [8, 9, 10, 11, 12].

A related modification of DAT controlled by PKC is palmitoylation, the reversible adduction of a lipid moiety to sulfhydryl groups of cytoplasmically oriented cysteines. In rat (r) and human (h) transporters, palmitate incorporation occurs on multiple sites, including Cys580/581 at the interface of the intracellular end of transmembrane domain 12 (TM12) and the cytoplasmic C‐terminus [13]. Palmitoylation increases DA transport velocity, opposes PKC‐induced down‐regulation, and is suppressed by activation of PKC [13, 14, 15]. These characteristics are opposite those of transporter phosphorylation, and the modifications thus function synergistically to integrate incoming information in control of reuptake.

In this study we now show that DAT palmitoylation is also mechanistically linked to METH. For these studies, we utilized a single high‐dose injection model in rats that has been extensively characterized by Fleckenstein and colleagues to investigate mechanisms of METH‐induced transport down‐regulation [16, 17]. These studies showed that METH, but not cocaine, induces transport reductions that are rapid, reversible, and not due to changes in DAT protein levels, consistent with a post‐translational mechanism. Related in vitro studies demonstrating transport down‐regulation to METH in cultured cells or synaptosomes support that responses do not require neuronal circuitry or input from DA receptors and are PKC‐dependent [8, 16, 17, 18, 19, 20, 21].

Using this injection paradigm, we now show that palmitoylation of striatal transporters undergoes rapid and transient reductions in response to METH, which to the best of our knowledge is the first demonstration of in vivo reversibility of a DAT post‐translational modification. Palmitoylation reductions occurred within minutes of METH injection, similar to the time course of transport down‐regulation, and in model cells palmitoylation‐deficient C580A DAT displayed a greater magnitude of METH‐induced transport down‐regulation than WT DAT. These findings, in conjunction with other similarities including PKC dependency and lack of cocaine effect, support that palmitoylation functions to oppose entry of DAT into METH‐induced down‐regulated states. Recovery of transport was markedly slower than repalmitoylation; however, potentially indicating that palmitoylation status exerts minimal effect on reversal of down‐regulation processes. These findings support palmitoylation as a rapid response mechanism that modulates METH impacts on DAT and suggest palmitoylation more broadly as a regulator of reuptake in additional physiological and pathophysiological conditions.

## 2 Materials and Methods

### 2.1 Materials

[7,8‐^3^H]DA (45 Ci/mmol) was from ViTrax (Placentia, CA, USA); DAT monoclonal antibody 16 (MAb 16) was previously authenticated [22] and available commercially (Invitrogen‐ThermoFisher MA5‐24796); N‐ethylmaleimide (NEM), high‐capacity NeutrAvidin‐agarose resin, and bicinchoninic acid (BCA) protein assay reagent were from ThermoFisher Scientific (Waltham, MA, USA); HPDP‐biotin was from APExBIO (Houston, TX, USA); (−)‐Cocaine, AMPH, METH, DA, and other fine chemicals were from MilliporeSigma (Sheboygan Falls, WI, USA). Bisindolylmaleimide I (BIM I) was from Cayman Chemical (Ann Arbor, MI, USA). Rats were purchased from Envigo (Lafayette, IN, USA). All animals were housed and treated in accordance with regulations established by the National Institutes of Health and approved by the University of North Dakota Institutional Animal Care and Use Committee.

### 2.2 Drug Injection and Membrane/Synaptosome Preparation

Male Sprague–Dawley rats (175–300 g) were given single 1 mL subcutaneous injections of saline, METH, or (−)‐cocaine to achieve final drug dosages of 15 mg/kg. Animals were decapitated at indicated times after injection, and striata were rapidly removed, weighed, and placed in ice‐cold sucrose phosphate buffer (SP, 0.32 M sucrose, 10 mM Na_2_PO_4_, pH 7.4). To prepare synaptosomes, tissue was homogenized at 4°C in 2 mL of SP buffer using a glass/Teflon homogenizing apparatus, volume brought up to 10 mL, and homogenates centrifuged at 3000 × g for 3 min at 4°C to remove nuclei and debris. The supernatant fraction was transferred to a fresh tube and centrifuged at 17 000 × g for 12 min. The pellet was washed two times by resuspension in 5 mL of SP buffer and centrifugation at 17 000 × g for 12 min at 4°C. The final pellet was resuspended with SP buffer at 15 mg/mL original striatal wet weight, and synaptosomes were analyzed for [^3^H]DA uptake or DAT immunoblotting. For analysis of palmitoylation, striatal membranes were prepared by homogenization of striata in SP buffer containing 100 mM EDTA (SP+) using a Polytron tissue homogenizer and centrifugation at 1000 × g for 10 min at 4°C to pellet nuclei and debris. The supernatant fraction was transferred to a fresh tube and centrifuged at 12 000 × g for 12 min, and the pellet was resuspended in 5 mL of SP+ buffer and centrifuged at 12 000 × g for 12 min at 4°C. The final membrane pellet was resuspended with SP+ buffer at a final concentration of 50 mg/mL original striatal wet weight and used for palmitoylation analysis via ABE.

### 2.3 Cell Culture and Treatments

Lilly laboratory porcine kidney (LLC‐PK_1_) cells expressing rDAT were grown in alpha minimum essential medium (AMEM) supplemented with 5% fetal bovine serum, 2 mM L‐glutamine, 200 μg/mL G418, 100 μg/mL penicillin/streptomycin, and 0.25 μg/mL amphotericin B and maintained in a humidified 5% CO_2_ environment at 37°C. For analysis of METH, AMPH, cocaine, or BIM I effects, cells were treated for the times or combinations described in the text and figure legends with vehicle, 10 μM METH, 10 μM AMPH, 10 μM (−)‐cocaine, or 10 μM BIM I. For BIM I treatments, cells were preincubated with BIM I for 5 min prior to the addition of METH and continued incubation for 30 additional min. At the end of the treatment time, the medium was removed, cells were washed with the indicated buffer, and analyzed for DAT palmitoylation or [^3^H]DA uptake as described below.

### 2.4 [ ^3^H]DA Uptake Analysis

Uptake in synaptosomes was initiated by the addition of 100 μL of synaptosomes to 900 μL of modified Krebs phosphate buffer (MKP, 16 mM potassium phosphate, 126 mM NaCl, 4.8 mM KCl, 1.4 mM MgSO_4_, 10 mM glucose, 1.1 mM ascorbic acid, and 1.3 mM CaCl_2_, pH 7.4), bringing the final concentration of [^3^H]DA to 10 nM and total DA to 1 μM. For DA saturation analyses, total DA concentrations were between 0.3 and 1 μM containing 10 nM [^3^H]DA. Assay tubes were incubated for 5 min with shaking at 30°C, and transport was stopped by the addition of 5 mL ice‐cold SP buffer and filtration through a glass/fiber filter using a Brandel filtration device. Radioactivity remaining on washed filters was measured by liquid scintillation counting. Nonspecific uptake was determined by the addition of 100 μM (−)‐cocaine and subtracted from total uptake to determine specific uptake values. *K*_m_ and *V*_max_ constants were determined by nonlinear regression analysis using GraphPad Prism Software. For analyses in cells, WT or mutant rDAT LLC‐PK_1_ cells were grown in 24‐well plates to 80% confluence and rinsed twice with 0.5 mL of 37°C KRH buffer. Cells were then incubated with vehicle, 10 μM METH, or 10 μM (−)‐cocaine in Krebs‐Ringer/HEPES buffer (KRH: 25 mM HEPES, 125 mM NaCl, 4.8 mM KCl, 1.2 mM KH_2_PO_4_, 1.3 mM CaCl_2_, 1.2 mM MgSO_4_, 5.6 mM glucose, pH 7.4) buffer for the indicated times. For washout studies after METH treatment, cells were rapidly washed with 37°C KRH to remove the drug, followed by incubation with KRH for the indicated times at 37°C. DA uptake assays were initiated by the addition of 10 μL of a 50× DA stock solution to bring the final concentration of [^3^H]DA to 10 nM and total DA to 3 μM. Uptake was conducted for 8 min and terminated by rapidly washing the cells two times with ice‐cold KRH buffer. Nonspecific uptake was determined by the addition of 100 μM (−)‐cocaine to parallel wells and subtracted from total uptake values to determine specific uptake. Cells were lysed with 1% Triton X‐100, and radioactivity in the lysates was measured by liquid scintillation counting.

### 2.5 Cell Membrane Preparation

Cells were grown to approximately 90% confluency in 15 cm cell culture dishes, treated with the indicated drug or vehicle for the indicated time, media were removed, and cells were washed twice with ice‐cold Buffer B (0.25 M sucrose, 10 mM triethanolamine, pH adjusted to 7.8 with 100 mM acetic acid, and supplemented with 1 μM phenylmethylsulphonyl (PMSF) and 5 μM ethylenediaminetetraacetic acid (EDTA)). For washout studies, cells were rapidly washed with 37°C KRH buffer to remove the drug. After the initial rapid wash, the cells were incubated with KRH for the indicated times at 37°C. The cells were then placed on ice and washed twice with ice‐cold Buffer B. The cells were then scraped using Buffer B, collected in 1.7 mL microcentrifuge tubes, and centrifuged at 3000 × g for 5 min at 4°C. The supernatant was removed, and the cell pellet gently resuspended in Buffer C (0.25 M sucrose, 10 mM triethanolamine, 1 mM EDTA, pH 7.8 with 100 mM acetic acid, supplemented with 1 μM PMSF and 5 μM EDTA), transferred to a Dounce homogenizer and homogenized with 30 up and down strokes. The sample was then centrifuged for 10 min at 800 × g to remove nuclei and cell debris. The supernatant was then collected and centrifuged at 16 000 × g for 12 min, and the resulting membrane pellet was resuspended in 1 mL SP buffer (0.32 M sucrose, 10 mM sodium phosphate, pH 7.4 with 1 μM PMSF and 5 μM EDTA), assayed for protein concentration, and stored at −20°C for analysis.

### 2.6 Acyl‐Biotin Exchange

Palmitoylated proteins were detected using procedures adapted from Wan et al. [23]. (i) To block free cysteine thiol groups on DAT, cell membranes (200–300 μg protein) were solubilized in 250 μL lysis buffer (50 mM HEPES pH 7.0, 2% SDS (w/v), 1 mM EDTA) containing 25 mM NEM and incubated for 20 min in a 37°C water bath followed by end‐over‐end mixing for at least 1 h at ambient temperature. Proteins were precipitated by the addition of 1 mL acetone and mixing, followed by centrifugation at 18 000 × g for 10 min. The protein pellet was resuspended in 250 μL lysis buffer containing 25 mM NEM and incubated for an hour at ambient temperature with end‐over‐end mixing. This process was repeated a final time with overnight incubation and end‐over‐end mixing. NEM was removed by acetone precipitation and centrifugation, and the protein pellet was resuspended in 250 μL 4SB buffer (50 mM Tris, 5 mM EDTA, 4% SDS, pH 7.4). Final protein pellets were resuspended in 200 μL 4SB buffer. (ii) Endogenous thioester‐linked palmitoyl groups on DAT were removed by treatment with hydroxylamine (HA), using treatment of parallel aliquots with Tris–HCl buffer as a negative control to verify NEM blockade of free sulfhydryl groups. The resuspended protein sample was split into equal volumes (100 μL) that were treated with 50 mM Tris–HCl, pH 7.4 or 0.7 mM HA in Tris buffer adjusted to pH 7.4 and incubated at room temperature for 30 min with end‐over‐end mixing. (iii) Both samples were then treated with sulfhydryl‐specific HPDP‐Biotin (0.4 mM final, 50 mM Tris–HCl, pH 7.4) for 1 h at room temperature with end‐over‐end mixing to biotinylate free sulfhydryl groups liberated by HA treatment. HA and unbound biotin were removed with three sequential acetone precipitations (centrifugation at 18 000 × g for 10 min, supernatant fraction removal, and resuspension of the protein pellet in 250 μL 4SB) followed by resuspension/solubilization of the final protein pellet in 75 μL of lysis buffer. (iv) For determination of total DAT content in the ABE sample, a 10 μL aliquot was set aside for immunoblotting while 65 μL was diluted in 1500 μL Tris buffer and incubated with 50 μL of a 50% slurry of high‐capacity NeutrAvidin resin to affinity purify the biotinylated proteins overnight at 4°C with end‐over‐end mixing. Unbound proteins were removed by three cycles of 8000 × g centrifugation, removal of the supernatant fraction, and resuspension in 750 μL radioimmunoprecipitation assay buffer (RIPA: 1% Tx‐100, 1% sodium deoxycholate, 0.1% SDS, 125 mM sodium phosphate, 150 mM NaCl, 2 mM EDTA, 50 mM NaF). Proteins were eluted from the final pellet by incubation in 2× Laemmli sample buffer (SB: 125 mM Tris–HCl, 20% glycerol, 4% SDS, 200 mM DTT, 0.005% bromophenol blue) for 20 min at ambient temperature. Eluted samples were then subjected to SDS‐PAGE and immunoblotted for rDAT with mouse MAb16 primary antibody.

### 2.7 Quantification of DAT Palmitoylation by ABE

Palmitoylation of DAT was quantified as previously described [15, 24, 25]. Briefly, aliquots (10 μL) taken from final resuspension (75 μL) just prior to NeutrAvidin extraction in the ABE assay were used to directly assess total DAT levels in the sample and normalize DAT palmitoylation levels in this same sample for comparison to other samples and treatments. Palmitoylated DAT band intensities were quantified using Quantity One software (Bio‐Rad), normalized to total DAT protein present, and expressed as % control. Bands in immunoblots represent 13.3% of the total and 86.7% of the palmitoylated DAT in each sample. Control values were set to 100%.

### 2.8 Immunoblotting

Samples were resolved using 4%–20% SDS‐polyacrylamide gels, transferred to PVDF membranes, and assayed for DAT as previously described [22]. Membranes were probed using a 1:1000 dilution of mouse monoclonal N‐terminal Ab16 (MAb16) for rDAT (ThermoFisher Scientific). Anti‐mouse alkaline phosphatase‐conjugated secondary antibody was utilized along with ImmunStar (Bio‐Rad) substrate and the Bio‐Rad gel documentation system to visualize the blots and quantify levels of immunostaining in the linear range using Quantity One software (Bio‐Rad).

### 2.9 METH Extraction and LC–MS/MS Analysis

Synaptosome samples (25 μL, *n* = 6) taken 30 min after METH injection were spiked with a deuterium‐labeled METH as an internal standard (100 pg. methamphetamine‐d_5_) followed by methanol extraction by addition of 75 μL methanol, thorough mixing, and incubation in a sonic water bath for 1 min, followed by centrifugation at 20 000 × g for 10 min at room temperature. The supernatant fraction was transferred to a sample vial. Five μL of sample was loaded onto an ACQUITY UPLC HSS T3 column (1.8 μM, 100 Å pore diameter, 2.1 × 150 mm) with an ACQUITY UPLC HSS T3 precolumn (1.8 μM, 100 Å pore diameter, 2.1 × 5 mm, Waters, Milford, MA), and METH was resolved using a Waters Acquity UPLC system (Waters, Milford, MA). A gradient elution with solvent A (0.1% formic acid in water) and solvent B (0.1% formic acid in acetonitrile) at 0.3 mL/min was used. Initial 10% B was maintained for 0.5 min, then increased to 40% over 3.5 min. At 6.5 min, %B was increased to 75%, and at 9 min returned to 10%. The column was equilibrated with 10% B for 3 min between injections. MS/MS analysis was performed on a Waters Xevo TQ‐S triple quadrupole mass spectrometer (Waters, Milford, MA) using multiple reaction monitoring mode. MS was operated in a positive ESI mode. METH was quantified against the deuterium‐labeled internal standard METH‐d_5_ based on the calibration curve, and the mean values were used to calculate the residual METH in the assay tube. The following mass transitions (with collision energies indicated in parentheses, V) were used: for METH‐d_5_–155.18/120.96 (10, for confirmation) and 155.18/91.73 (16, for quantification); for METH—150.15/118.97 (10, for confirmation) and 150.15/90.96–85.37/95.10 (16, for quantification). MassLynx 4.1 (Waters) was used for instrument control and data processing.

## 3 Results

**FIGURE 1:**
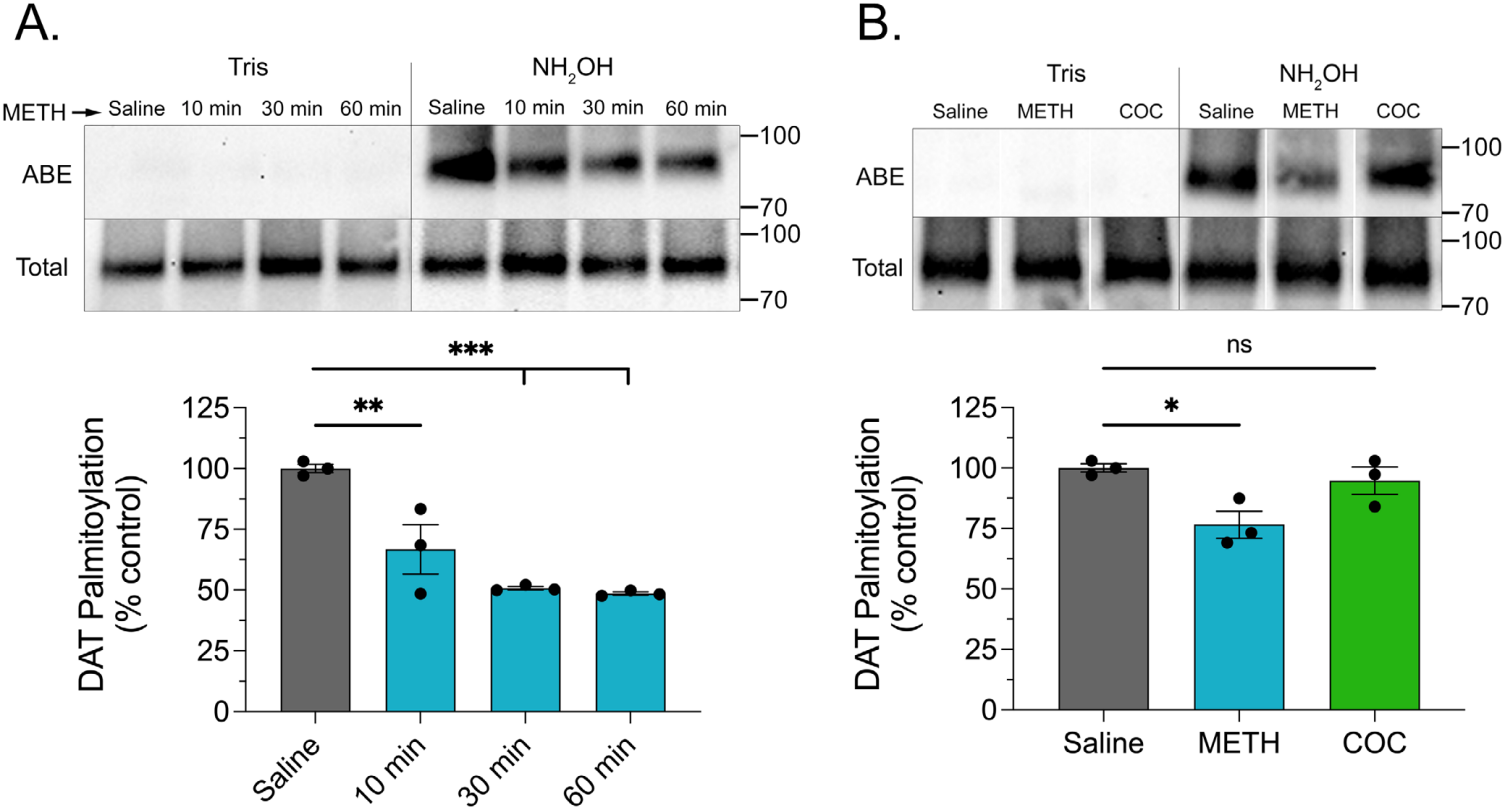
DAT palmitoylation is reduced by in vivo METH. Male Sprague–Dawley rats were given s.c. injections of saline, METH (15 mg/kg), or (−)‐cocaine (15 mg/kg) and sacrificed at indicated times (A) or after 30 min (B). Striatal tissue was isolated and analyzed by ABE for DAT palmitoylation (upper rows) or by immunoblot for total DAT (lower rows). Blots show ABE, Tris specificity controls, and total DAT analyses from representative experiments, and histograms show quantification of DAT palmitoylation normalized for total DAT protein and expressed as a fraction of saline control set to 100% (means ± S.E. of 3 independent experiments performed in duplicate). *p < 0.05; **p < 0.01; ***p < 0.001 vs. control; one‐way ANOVA with Tukey's post hoc test. ns, not significant. White spaces between lanes in panel B indicate cropping to remove duplicate samples.

To determine the effects of in vivo METH or (−)‐cocaine on DAT palmitoylation, male Sprague–Dawley rats were given single s.c. injections of saline, 15 mg/kg METH, or 15 mg/kg (−)‐cocaine, sacrificed between 10 and 60 min post‐injection, and striatal transporters were analyzed for palmitoylation using the acyl biotinyl exchange (ABE) assay (Figure 1). In this method, endogenous palmitate groups are chemically replaced with a biotinylated moiety that allows for extraction and quantitative immunoblotting of originally palmitoylated forms, permitting assessment of real‐time in vivo changes in modification levels. Figure 1A shows that within 10 min of METH injection, DAT palmitoylation was reduced to 66.8% ± 10.1% of control levels (*p* < 0.01) and remained suppressed through 30 min (50.8% ± 0.7% of control, *p* < 0.001) and 60 min (48.6% ± 0.7% of control, *p* < 0.001). In separate experiments, we investigated uptake blocker actions using (−)‐cocaine and found that 30 min after injection, DAT palmitoylation was unchanged (94.8% ± 5.6% of control, *p* > 0.05) compared to that of rats given METH in parallel, which showed reductions to 76.6% ± 5.5% of control (*p* < 0.05) (Figure 1B). Equivalent levels of total DAT were present in all ABE samples, indicating that palmitoylation reductions are due to enzymatic alterations and not to changes in DAT expression.

**FIGURE 2:**
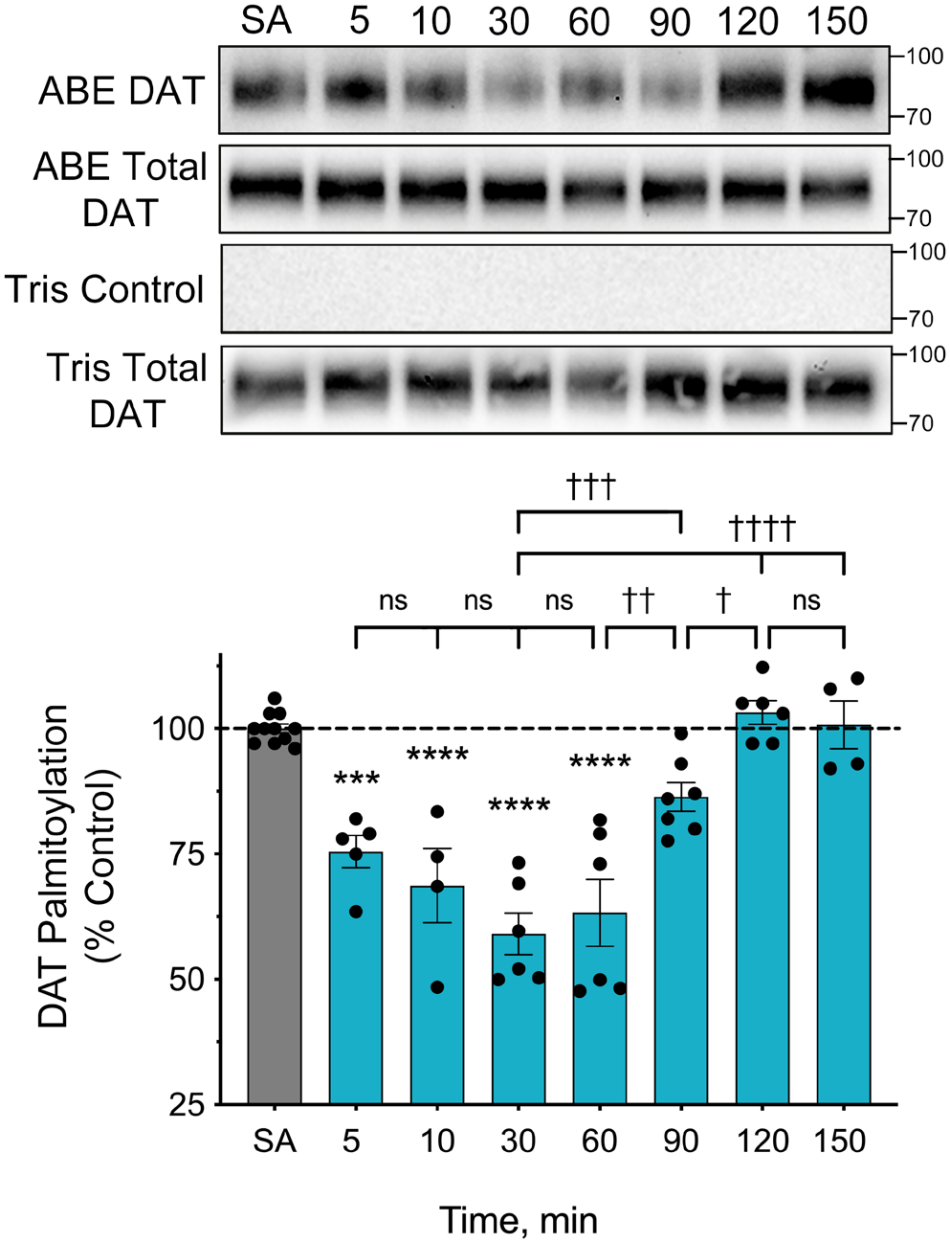
Onset and reversibility of DAT palmitoylation reductions to in vivo METH. Male Sprague–Dawley rats were given s.c. injections of saline (SA) or METH (15 mg/kg), sacrificed at the indicated times, and striatal membranes analyzed for DAT palmitoylation. The blots show ABE (top row), corresponding total DAT (second row), and ABE specificity controls (bottom two rows) from a representative experiment. Histogram shows quantification of DAT palmitoylation normalized for total DAT expressed as a fraction of saline control set to 100% (means ± S.E. of 4–7 experiments). ***p < 0.001; ****p < 0.0001; indicated time points vs. SA control; ^†^ *p* < 0.05; ^††^ *p* < 0.01; ^†††^ *p* < 0.001; ^††††^ *p* < 0.0001, confidence values between indicated time points. ns, not significant, (one‐way ANOVA with Tukey's post hoc test).

To further characterize the response, we analyzed the additional time points shown in Figure 2. Here, rats were given s.c. injections of saline or 15 mg/kg METH and sacrificed between 5 and 150 min post‐injection. Within 5 min, DAT palmitoylation was reduced to 75.5% ± 3.2% of control, with decreases maintained through 10 min (68.7% ± 7.4% of control), 30 min (59.0% ± 4.1% of control), and 60 min (63.3% ± 6.7% of control) (all *p* < 0.001 or *p* < 0.0001). At later time points, palmitoylation gradually returned to starting levels, reaching values of 86.4% ± 2.8% of control at 90 min, 103.2% ± 2.3% of control at 120 min, and 100.7% ± 4.8% of control at 150 min (all *p* > 0.05 vs. control and *p* < 0.001–0.0001 vs. palmitoylation level at 30 min).

**FIGURE 3:**
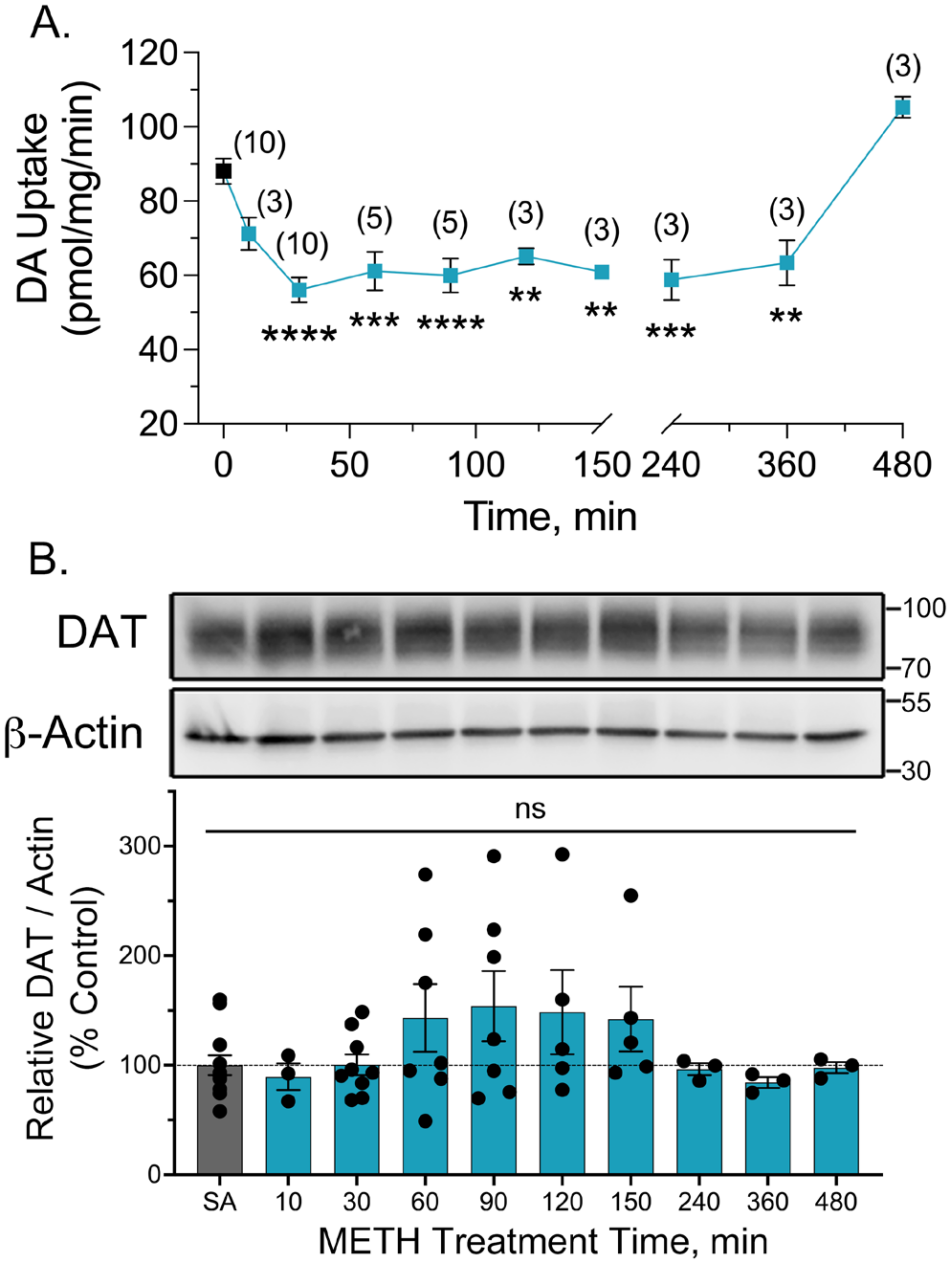
Onset and reversibility of DA transport reductions in synaptosomes from METH‐treated rats. Male Sprague–Dawley rats were given s.c. injections of saline or METH (15 mg/kg) and sacrificed at the indicated time points. Synaptosomes were prepared from striatal tissue collected at each time point and equal amounts assessed for [^3^H]DA uptake or immunoblotted for total DAT. (A) DA transport at indicated times after injection (means ± S.E. of 3–10 experiments performed in quadruplicate; number of independent experiments indicated in parentheses). **p < 0.01, ***p < 0.001, ****p < 0.0001 relative to control (one‐way ANOVA with Tukey's post hoc test). (B) Representative immunoblots of total DAT and β‐Actin from aliquots of synaptosomes utilized for uptake. Histogram shows quantification of DAT normalized to β‐Actin for each time point (means ± S.E. of 4–7 experiments). All values *p* > 0.05 vs. control, ns, not significant. (one‐way ANOVA with Tukey's post hoc test).

We then examined the response of transport activity to METH treatment across the time points in which palmitoylation showed suppression and recovery (Figure 3). For these studies, rats were given s.c. injections of saline or 15 mg/kg METH and sacrificed 10–150 min after injection. Synaptosomes were prepared from striatal tissue and washed two times during preparation, which was previously demonstrated to reduce residual METH to levels that do not pharmacologically inhibit uptake [20]. We also used LC–MS/MS to quantify METH levels, and in six independent samples collected 30 min after injection, confirmed amounts to be consistent with assay concentrations of 0.4–3 nM, below the pharmacological threshold for uptake inhibition.

As previously described [16, 17], METH injection induced a rapid decrease in synaptosomal [^3^H]DA uptake, with a downward trend at 10 min and reductions to ~50%–60% of control values reached between 30 and 150 min post‐injection (*p* < 0.01–0.0001 vs. control) (Figure 3A). Previous characterization of this response demonstrated that transport activity remained suppressed for 3 h and returned to control levels by 24 h [16, 17]. Here we refined the recovery time, showing that activity remained suppressed between 4 and 6 h after injection and returned to control levels by 8 h (Figure 3A). Immunoblots of synaptosomes from these time points showed no changes in DAT protein levels (Figure 3B), indicating that transport reductions did not occur via loss of DAT protein.

**FIGURE 4:**
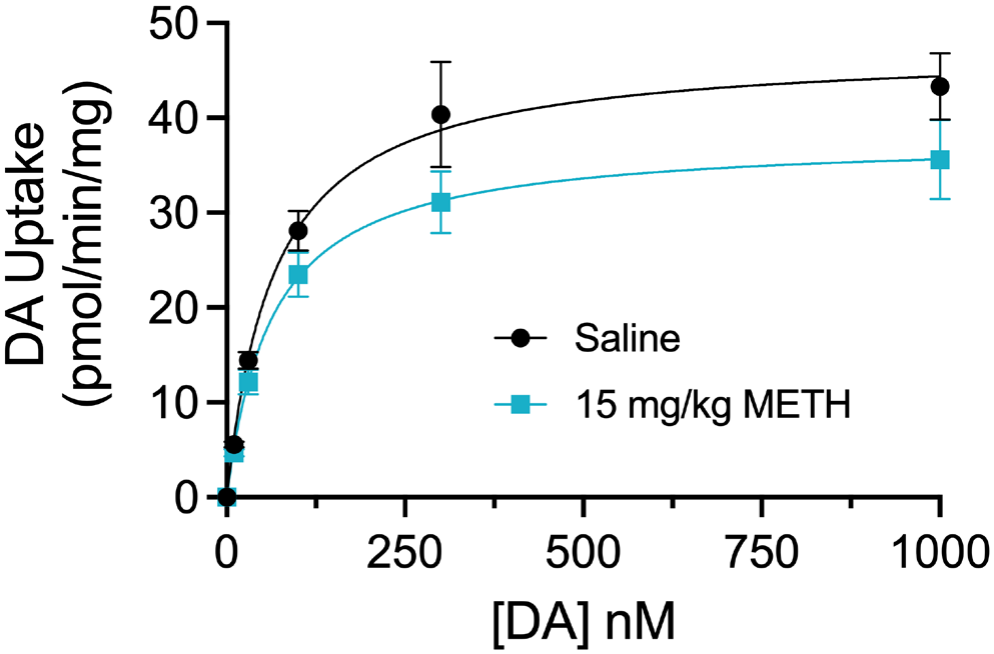
Kinetic analysis of DA transport activity after METH. Male Sprague–Dawley rats were given s.c. injections of saline or METH (15 mg/kg), sacrificed at 30 min, and synaptosomes subjected to DA transport saturation analysis. Curves show uptake activity (means ± S.E. from four independent experiments performed in triplicate). *V*_max_ and *K*_m_ values were determined in each experiment by nonlinear regression analysis using Michaelis–Menten kinetics with GraphPad Prism software. Mean *V*_max_ values were saline 47.4 ± 5.0 pmol/min/mg; METH 38.0 ± 4.4 pmol/min/mg (*p* < 0.05 vs. control); mean *K*_m_ values were: saline 66.5 ± 3.6; METH 63.7 ± 4.7 nM (not significant).

The mechanism of transport reduction in these conditions was assessed via DA transport saturation analysis using synaptosomes prepared from striatal tissue collected 30 min after injection (Figure 4). Results show that transport losses were mediated by reductions in *V*_max_ from 47.4 ± 5.0 pmol/min/mg in synaptosomes from control animals to 38.0 ± 4.4 pmol/min/mg in synaptosomes from METH‐treated animals (*p* < 0.05), whereas there was no significant difference in *K*_m,DA_ between control (66.5 ± 3.6 nM) and METH conditions (63.7 ± 4.7 nM).

**FIGURE 5:**
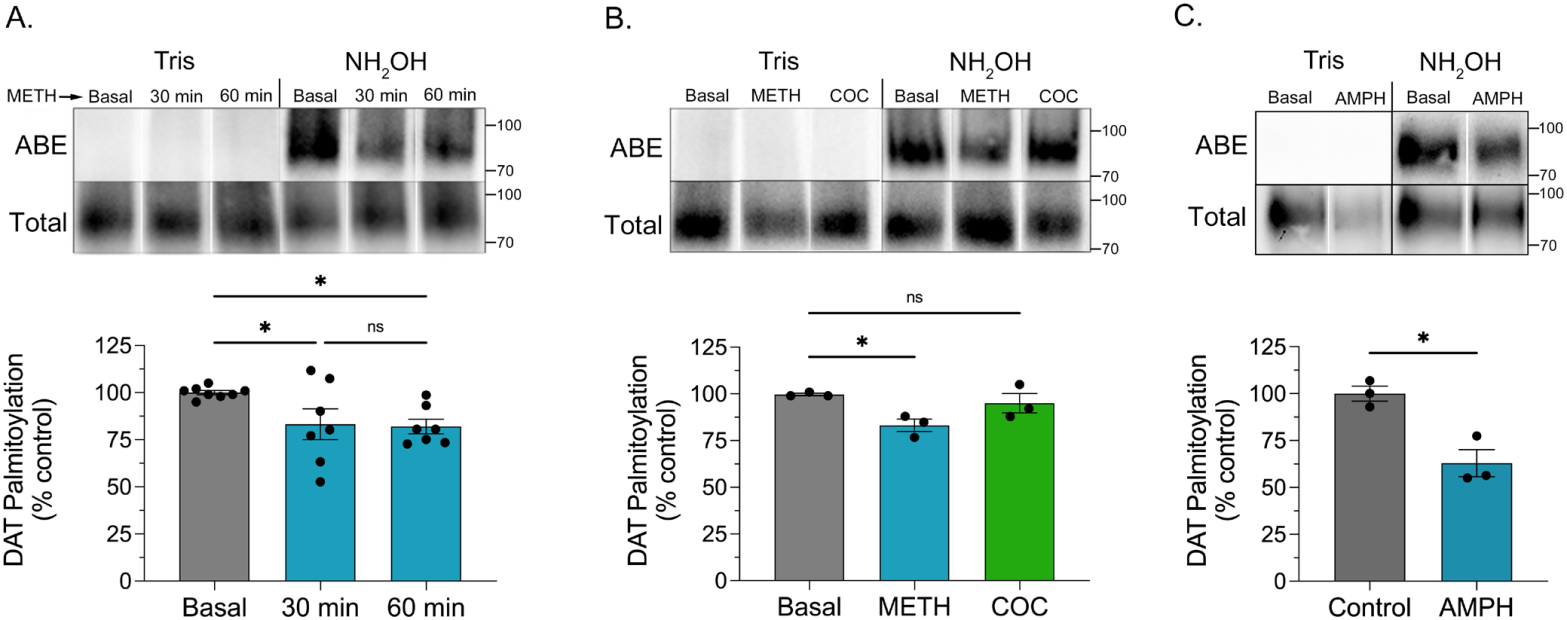
DAT palmitoylation is reduced by in vitro METH and AMPH. rDAT‐LLCPK_1_ cells were treated with vehicle or METH (10 μM) for the indicated times (A), with (−)‐cocaine (10 μM) or METH (10 μM) for 30 min (B), or with AMPH (10 μM) for 30 min (C), and membranes analyzed for DAT palmitoylation. Blots show representative ABE and Tris specificity controls (upper rows) and total DAT analyses (lower rows), and histograms show quantification of DAT palmitoylation normalized for total DAT and expressed as a fraction of vehicle control set to 100% (means ± S.E. of 3–7 independent experiments performed in duplicate). (A and B) *p < 0.05 vs. indicated treatment groups (one‐way ANOVA with Tukey's post hoc test). ns, not significant; (C) *p < 0.05 vs. indicated treatment groups (Student's *t*‐test). White spaces between lanes indicate removal of duplicate samples from the same experiment.

We then examined the effects of in vitro METH on transporter palmitoylation using rDAT‐LLCPK_1_ cells, which have been previously used to characterize DAT transport and phosphorylation responses to METH and PKC [8, 26] (Figure 5). In cells treated with 10 μM METH for 30 or 60 min, DAT palmitoylation was reduced to 83.2% ± 8.2% and 82.1% ± 3.8% of control, respectively (both *p* < 0.05) (Figure 5A), whereas no reductions were seen in cells treated with 10 μM (−)‐cocaine for 30 min (95.0% ± 5.2% of control, *p* > 0.05) compared to reductions to 84.2% ± 4.1% of control by METH assessed in parallel (*p* < 0.05) (Figure 5B). Palmitoylation of DAT in this cell line was reduced to a similar level (63.0% ± 7.2% of control) by in vitro AMPH (Figure 5C), indicating this effect as common to other psychostimulant substrates.

**FIGURE 6:**
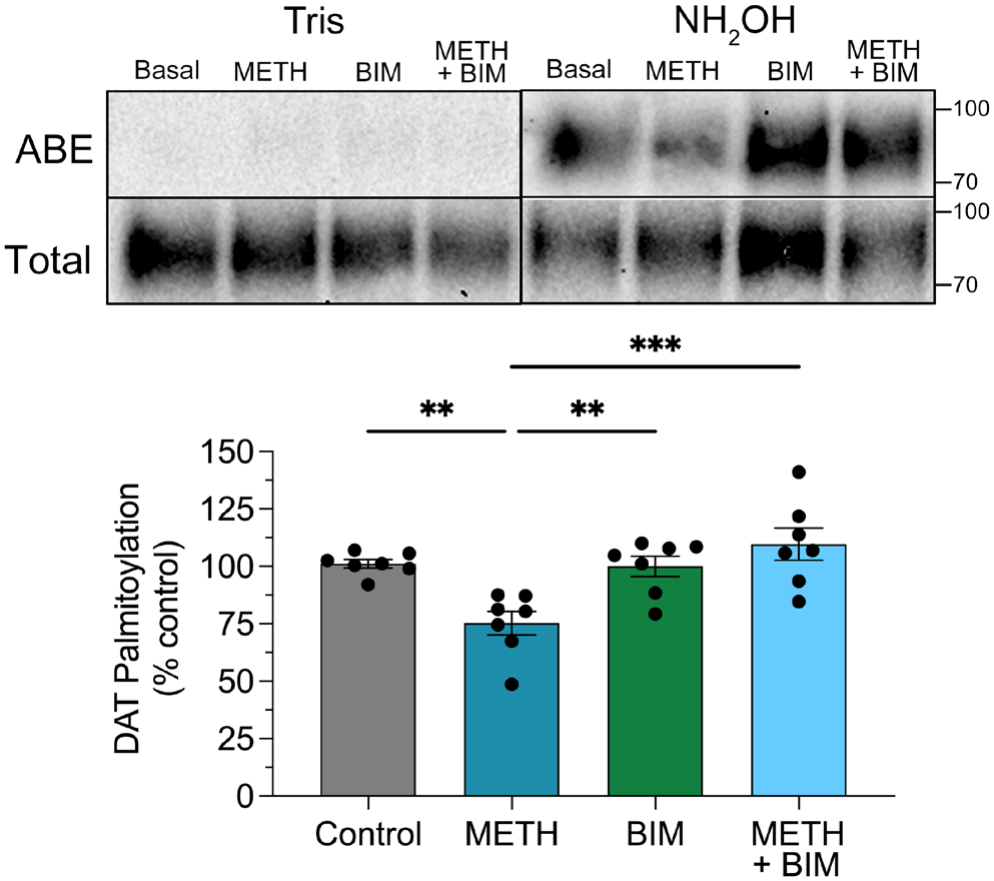
DAT palmitoylation reduced by in vitro METH is PKC‐dependent. rDAT‐LLCPK_1_ cells were treated as indicated with BIM (10 μM) for 5 min followed by indicated additions of vehicle or METH (10 μM) for an additional 30 min, and membranes analyzed for DAT palmitoylation. Blots show representative ABE and Tris specificity controls (upper rows) and total DAT analyses (lower rows), and histograms show quantification of DAT palmitoylation normalized for total DAT and expressed as a fraction of vehicle control set to 100% (means ± S.E. of seven independent experiments). **p < 0.01, ***p < 0.001 vs. indicated treatment groups, one‐way ANOVA with Tukey's post hoc test.

To test for involvement of PKC in these outcomes, we pre‐treated cells with the PKC inhibitor bisindolylmaleimide I (BIM) for 5 min followed by the addition of METH and continued incubation for 30 min (Figure 6). In these experiments, METH reduced DAT palmitoylation to 74.5% ± 5.1% of vehicle control (*p* < 0.01), 10 μM BIM produced no effect (99.3% ± 4.5% of control, *p* > 0.05), and the addition of 10 μM BIM prior to and during METH treatment prevented the reduction of palmitoylation (108.4% ± 6.9% of control, *p* > 0.05). This supports the involvement of PKC in METH suppression of palmitoylation, which is mechanistically and functionally consistent with our earlier findings that palmitoylation is suppressed by PKC activation [15] and that METH‐induced transport down‐regulation is PKC‐dependent [8].

**FIGURE 7:**
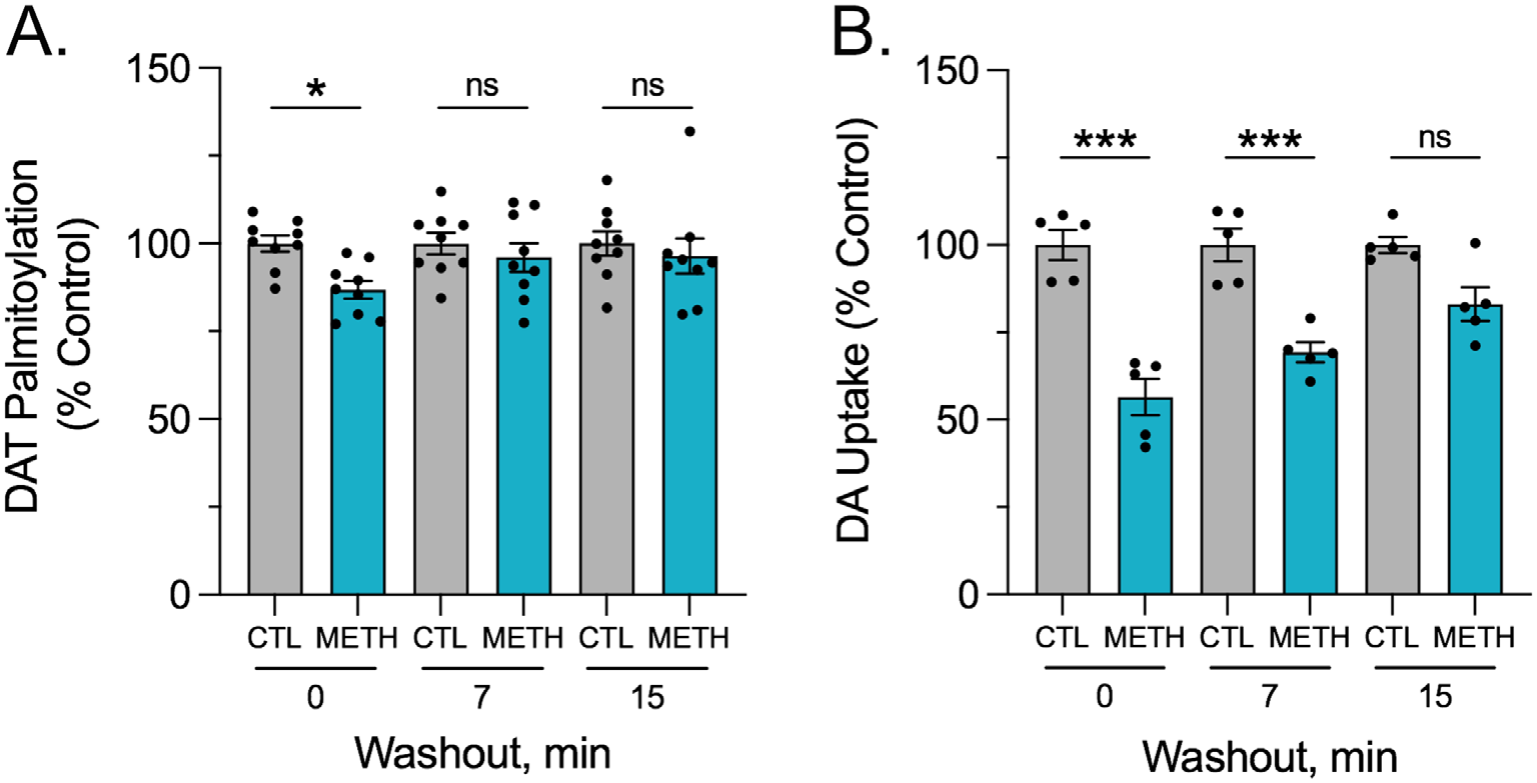
Reversibility of DAT palmitoylation and transport down‐regulation to in vitro METH. rDAT‐LLCPK_1_ cells were treated with vehicle (gray bars) or 10 μM METH (blue bars) for 30 min followed by washing to remove METH and incubation with buffer for the indicated times prior to analysis of palmitoylation or [^3^H]DA transport. (A) DAT palmitoylation normalized for total DAT protein at each time point and expressed as a fraction of treatment‐matched controls set to 100% (means ± SE of nine independent experiments). *p < 0.05 vs. control; one‐way ANOVA with Fisher's LSD test. (B) Transport activity expressed as a fraction of treatment‐matched controls set to 100% (means ± S.E. of five experiments performed in triplicate). ***p < 0.001; one‐way ANOVA with Tukey's post hoc test. ns, not significant.

To determine the reversibility of DAT palmitoylation and down‐regulation to in vitro METH, we performed wash‐out studies (Figure 7). Here, rDAT‐LLCPK_1_ cells were treated for 30 min with vehicle (gray bars) or 10 μM METH (blue bars), rapidly washed two times to remove the drug, and either assayed immediately or incubated with buffer for 7 or 15 min prior to assessment. In palmitoylation studies (Figure 7A), modification of DAT in vehicle‐treated cells was not changed in response to washing and incubations (all values *p* > 0.05 vs. 0 min control). In METH‐treated cells assayed immediately after washing, palmitoylation was reduced to 86.8% ± 7.5% of treatment‐matched control (*p* < 0.05), similar to findings in Figure 5. With buffer incubation after removal of the drug, palmitoylation recovered rapidly, returning to control levels within 7 min (96.0% ± 12.2% of control) and 15 min (96.5% ± 15.1%) (both *p* > 0.05 vs. treatment‐matched control).

Reversibility of in vitro METH effects on DA uptake is shown in Figure 7B. In cells that received vehicle treatment and washing (gray bars), transport activity showed no changes in magnitude at any time points (all values *p* > 0.05 vs. 0 min control). In METH‐treated cells assessed immediately after washing, transport was reduced to 63.1% ± 10.4% of treatment‐matched control (*p* < 0.001), similar to down‐regulation results seen in previous in vitro AMPH and METH pretreatment studies [8, 27]. In treated and washed cells given incubations prior to analysis, transport remained down‐regulated at 7 min (69.5% ± 5.6% of control, *p* < 0.001) but recovered to 82.8% ± 7.9% of control level by 15 min (*p* > 0.05) and to 100.9% ± 21.1% of control by 30 min (*p* > 0.05) (not shown).

**FIGURE 8:**
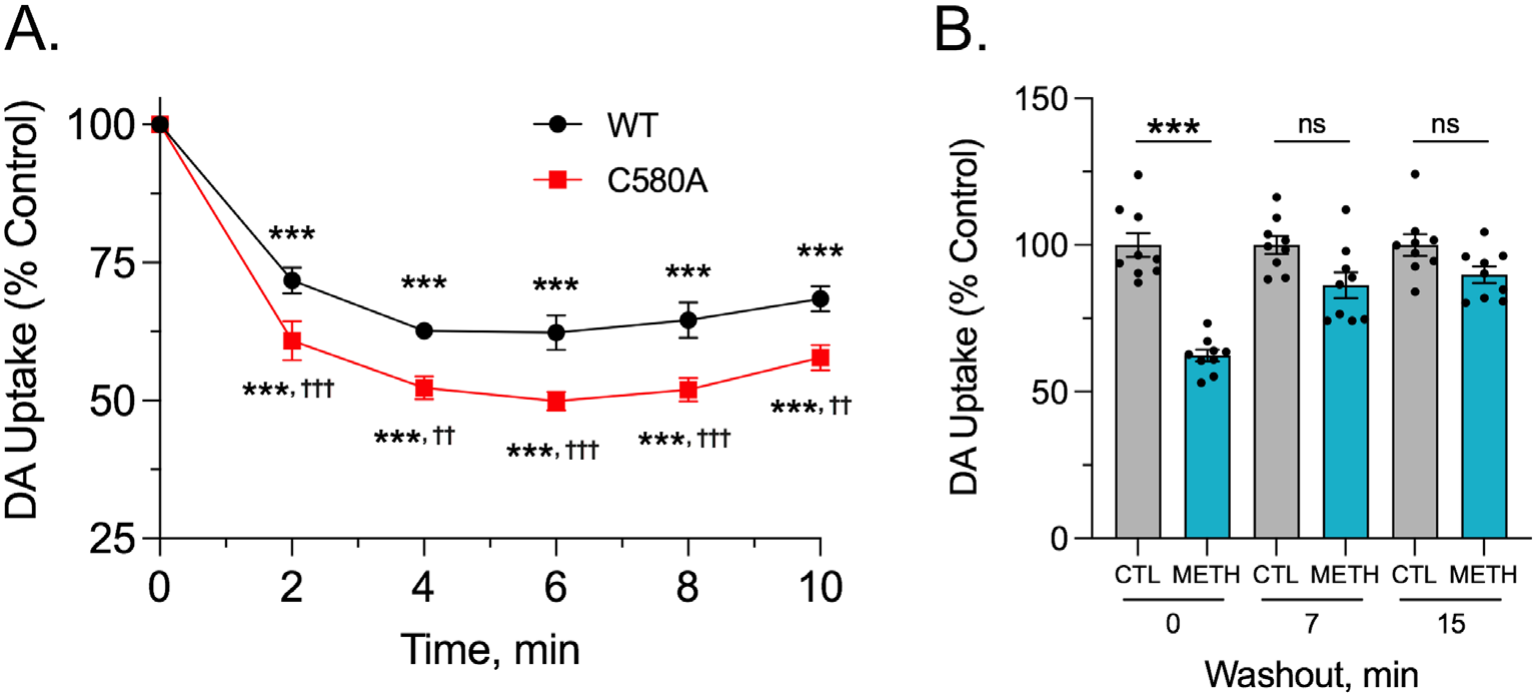
C580A DAT undergoes enhanced down‐regulation to METH and rapid reversibility after METH washout. (A) LLPCK_1_ cells expressing WT rDAT (black) or C580A rDAT (red) were treated with vehicle or 10 μM METH for the indicated times, washed to remove METH, and assayed for [^3^H]DA uptake. Symbols represent means ± S.E. of eight experiments performed in triplicate. ***p < 0.001 METH vs. respective vehicle controls for each form. ^††^ *p* < 0.01; ^†††^ *p* < 0.001; C580A DAT vs. WT DAT at matching time points, two‐way ANOVA with Tukey's post hoc test; significant main effects of time [F(5, 68) = 119.6, *p* < 0.0001] and DAT type [F(1, 14) = 36.48, *p* < 0.0001], as well as a significant time × DAT type interaction [F(5, 68) = 2.430, *p* = 0.0437]. (B) C580A‐rDAT‐LLCPK_1_ cells were treated with vehicle (gray bars) or 10 μM METH (blue bars) for 30 min followed by rapid washing and incubation with buffer for the indicated times prior to analysis of [^3^H]DA transport. Histogram shows transport activity of control and treated cells at each time point after washing (means ± S.E. of nine experiments performed in triplicate), expressed as a fraction of vehicle control for each time point set to 100%. ***p < 0.001; one‐way ANOVA with Tukey's post hoc test. ns, not significant.

About 50% of endogenous palmitate incorporation on rDAT occurs on residue Cys580 at the base of TM12, with modification of this site mechanistically linked to increased transport velocity and suppression of PKC‐induced down‐regulation [13]. To further investigate palmitoylation contributions to METH‐induced down‐regulation, we directly compared down‐regulation responses of C580A and WT DAT to METH treatment (Figure 8A). Here, WT‐ and C580A‐rDAT cells were treated in parallel with 10 μM METH for the indicated times, washed to remove the drug, and assayed for uptake. Similar to previous findings [8], transport activity of WT DAT displayed significant down‐regulation within 2 min of drug application, with activity plateauing at ~70% of starting levels (all values *p* < 0.001 vs. WT DAT control). In assays performed in parallel, transport activity of C580A DAT showed similar rapid reductions, with activity plateauing at ~50% of the C580A starting value (all values *p* < 0.001 vs. C580A DAT control). At all time points, the magnitude of C580A DAT down‐regulation was significantly greater than that of WT DAT (all values *p* < 0.01–0.001 vs. time‐matched WT DAT), supporting that METH‐induced transport down‐regulation is opposed by palmitoylation of Cys580.

The reversibility of METH‐induced C580A DAT down‐regulation was examined using the same washout paradigm as for WT DAT, with C580A DAT cells treated with vehicle or 10 μM METH for 30 min followed by washout and incubation prior to analysis of uptake activity (Figure 8B). Transport activity in cells given vehicle treatment showed no changes after washing and incubation (all *p* > 0.05 vs. 0 min control). In METH‐treated cells that were assayed immediately after washing, transport of C580A DAT showed reductions to 62.3% ± 6.0% of control (*p* < 0.001), similar to that shown in Figure 7A. In cells given buffer incubations after washing, uptake activity returned to control levels by 7 min (86.3% ± 13.1% of control) and 15 min (89.9% ± 8.4% of control) (both *p* > 0.05 vs. time‐matched control), indicating that C580A DAT transport activity recovered rapidly after removal of METH. Whether this rate of recovery differs from that of WT DAT was not directly investigated.

## 4 Discussion

The pathophysiological dysregulation of DAT by METH implicates the underlying processes in neurochemical outcomes and as potential targets for therapeutic intervention in drug addiction and other reuptake disorders. Here we now extend our understanding of METH mechanisms by demonstrating a previously unknown effect on transporter palmitoylation and its possible link to regulation and dysregulation of dopamine reuptake.

Our findings here show that DAT palmitoylation undergoes a rapid and transient reduction in response to METH in vivo and in vitro that shares time course, PKC dependency, and circuit/receptor‐independence characteristics with METH‐induced transport down‐regulation. In rats, striatal DAT palmitoylation was reduced within minutes of METH injection, remained suppressed for ~60 min, and returned to starting levels by 2 h. The rapid reduction of palmitoylation by METH closely correlates with drug entry into the brain, which occurs within minutes [28], and the return of palmitoylation to control levels could potentially follow from metabolic clearance of the drug, which in rats occurs with a T_½_ of ~1 h [29, 30].

In heterologous cells, DAT palmitoylation was also rapidly suppressed after METH addition, followed by a rapid return to starting levels after manual drug washout. Although the reductions in palmitoylation induced by in vitro METH were generally of lesser magnitude than found in vivo, the responses are qualitatively similar and support that impacts on palmitoylation can occur via direct actions on DAT that do not require neuronal circuitry or transmitter signaling. This interpretation is also consistent with findings that palmitoylation was not affected in vivo or in vitro by cocaine. This supports that under the conditions examined, changes to the modification are not induced by direct uptake blockade or cocaine‐induced elevations of in vivo DA, but whether circuitry or receptor mechanisms impact palmitoylation under other conditions remains to be investigated. The reduction and recovery of palmitoylation occurred without changes in DAT protein level, consistent with an enzymatic mechanism, and palmitoylation suppression in cells was prevented by a PKC inhibitor, suggesting a link between cytoplasmically transported METH, activation of PKC, and impacts on palmitoylation enzymes.

Because the velocity of DAT is enhanced by palmitoylation, these findings, in conjunction with the elevated down‐regulation of the palmitoylation‐deficient form, support that the modification serves to oppose the acute suppression of transport induced by METH that is associated in vivo with hyperdopaminergia. However, whereas palmitoylation and transport showed similar rates of reduction in response to METH injection, transport remained suppressed for hours after palmitoylation returned to starting levels. Additional events that mediate reduced reuptake thus remain in effect after palmitoylation has recovered and represent elements involved in the homeostatic restoration of transport after drug clearance.

**FIGURE 9:**
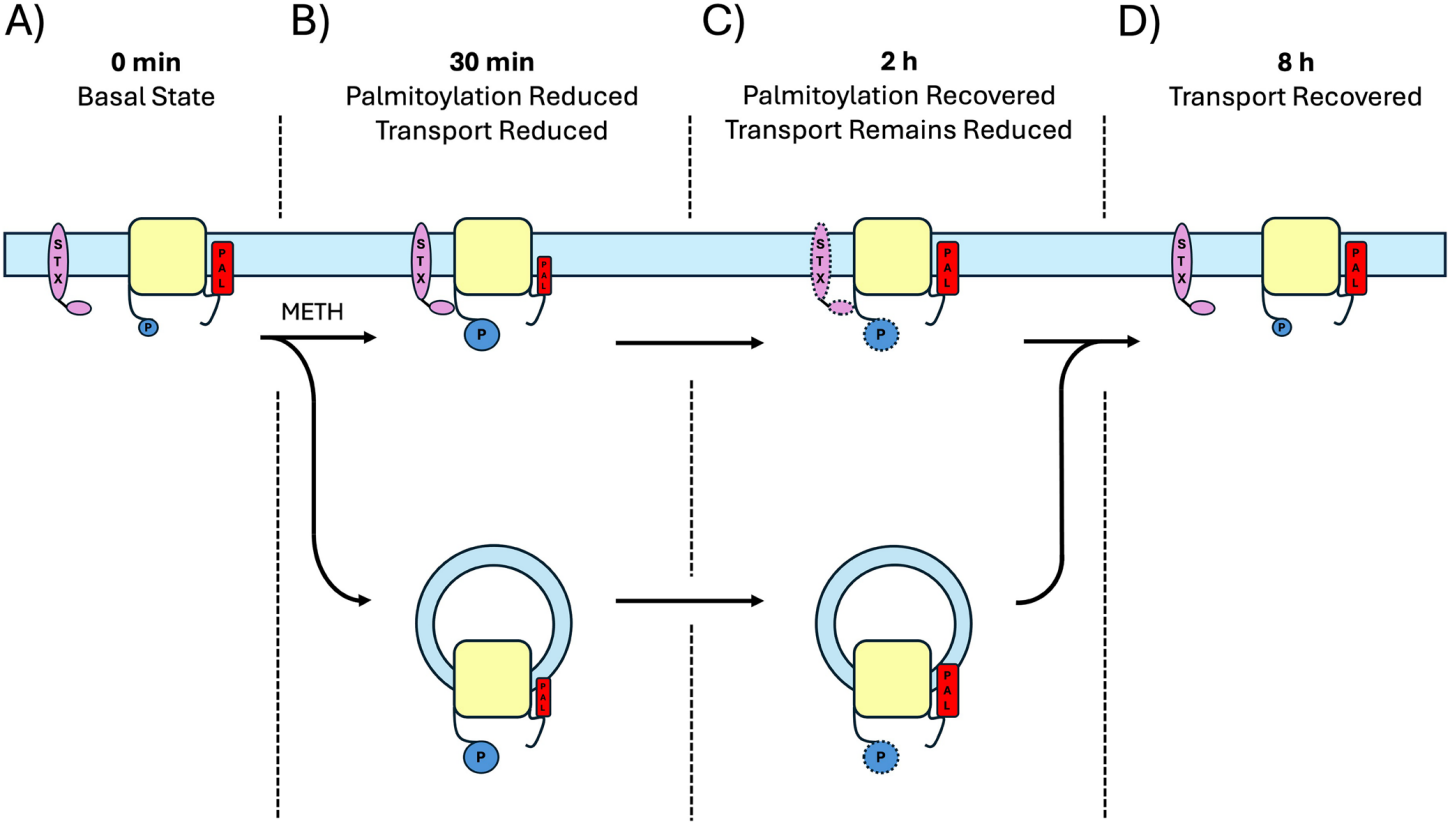
Model depicting potential events in METH‐induced responses of DAT palmitoylation and down‐regulation in rat striatum. DAT populations are shown in four functional and biochemical states prior to and after injection of METH. (A) In the basal state DAT is shown at the plasma membrane (blue line) with high levels of palmitoylation (red rectangles), low levels of phosphorylation (blue circles), and exhibiting minimal interactions with STX (purple oval). (B) 30 min after METH injection DAT palmitoylation is reduced and transport is down‐regulated. Potential mechanisms leading to reduced reuptake are increased transporter phosphorylation, increased interaction with STX, and/or removal of DAT from the surface via endocytosis (vesicles). For simplicity, other structural, interactome, or membrane mechanisms that could be involved with mediating down‐regulation as described in the text are not shown. (C) 2 h after METH injection, DAT palmitoylation has returned to initial levels but transport activity remains reduced. Events that could potentially underlie this condition are retention of high phosphorylation and/or‐STX interactions (indicated by stippling), or retention of endocytosed DAT in cytoplasmic vesicles. (D) 8 h after METH injection, DA transport has returned to initial levels. Events that could potentially underlie this response are return of transporter phosphorylation to initial levels, dissociation of DAT‐STX interactions, and return of endocytosed transporters to the plasma membrane.

Mechanistically, the suppression of down‐regulation by palmitoylation can be envisioned as the modification serving to stabilize higher velocity DAT forms or complexes such that reduced modification enables or enhances the transition of transporters into lower velocity states, as shown schematically in Figure 9. The precise mechanisms by which this might occur are not known, but down‐regulation of DAT by AMPH, METH, and PKC is mediated by multiple processes that could be impacted by palmitoylation, as described below. Rapid modulation of DA reuptake capacity is mediated by both kinetic control and reduction of plasma membrane transporter levels by endocytosis. These mechanisms may overlap and/or possess different onset and reversal rates that could relate to the dynamics of down‐regulation onset and recovery. In addition, whereas regulatory events induced by AMPH, METH, and PKC share many similarities, specific events may differ depending on stimulation conditions or systems analyzed [6, 31, 32, 33, 34, 35].

The regulatory property with the most current evidence for interfacing with both METH and palmitoylation is N‐terminal phosphorylation, which kinetically suppresses reuptake velocity of surface transporters [15], is stimulated within minutes of in vivo or in vitro METH [8, 15, 36], and is inhibited by palmitoylation [15]. Reciprocal regulation of phosphorylation by palmitoylation can occur in the absence of exogenous kinase or phosphatase modulation, supporting a direct connection between reduced palmitoylation and higher phosphorylation/lower velocity states.

A potentially congruent mechanism is the binding of the SNARE protein syntaxin 1A (STX) to DAT, which occurs through the transporter N‐terminal domain [37, 38]. In addition to regulating DAT channel and efflux properties [39, 40], STX suppresses DA transport velocity and stabilizes the tonic level of transporter phosphorylation [41]. The binding of STX to DAT is increased by AMPH [37], consistent with drug‐induced stimulation of transporter phosphorylation and down‐regulation. The impact of DAT palmitoylation on STX mechanisms has not yet been investigated, but STX is also palmitoylated [42], which modulates its membrane fusion properties and could potentially influence its DAT interactions or outcomes.

Palmitoylation also impacts multiple DAT membrane properties that have been associated with transport regulation and endocytosis. Computational modeling studies support that palmitoylation of hDAT Cys581 promotes structural conformations that enhance homodimerization [43], which has been linked to reuptake activity and membrane trafficking [34, 44, 45]. In addition, DAT lateral membrane mobility, which may influence microdomain targeting or regulome interactions [40, 46, 47], is reduced by palmitoylation and increased by phosphorylation, PKC, and AMPH [48]. For many proteins, palmitoylation also influences regulatory events related to membrane cholesterol [49], and although a role for palmitoylation of DAT in cholesterol events has not yet been directly examined, DAT kinetic properties, phosphorylation, and endocytosis have all been linked to direct cholesterol interactions and/or transporter partitioning into cholesterol‐rich membrane raft domains [50, 51, 52, 53].

Possible impacts of palmitoylation on the transporter C‐terminal domain are as yet speculative, but the location of Cys580/581 suggests the potential for the modification to alter TM12 properties such as conformation, tilt, hydrophobic matching, or protein–protein interactions. Such impacts could be propagated to the C‐terminus, including the adjacent juxtamembrane helix between residues 587–592 that is proposed to modulate transporter kinetics via interaction with intracellular loops residues [54]. This helix is also necessary for PKC‐stimulated endocytosis and binds the trafficking‐regulatory proteins G_βγ_ and Rit2 [55, 56, 57, 58], with a Rit2 homolog mediating AMPH behavioral sensitivity in *Drosophila* [59].

Less is known about the events that occur after METH clearance that underlie DAT resensitization and return of the reuptake system to homeostasis. The longer time needed for recovery of uptake compared to palmitoylation indicates that METH down‐regulation mechanisms reverse more slowly and may remain engaged with repalmitoylated transporters. This could occur via persistence of phosphorylation or STX binding with repalmitoylated surface transporters, or by endocytosed transporters undergoing repalmitoylation prior to plasma membrane recycling, as shown schematically in Figure 9, as well as by sustained involvement of repalmitoylated DATs with one or more of the other potential regulatory conditions described above.

**FIGURE 10:**
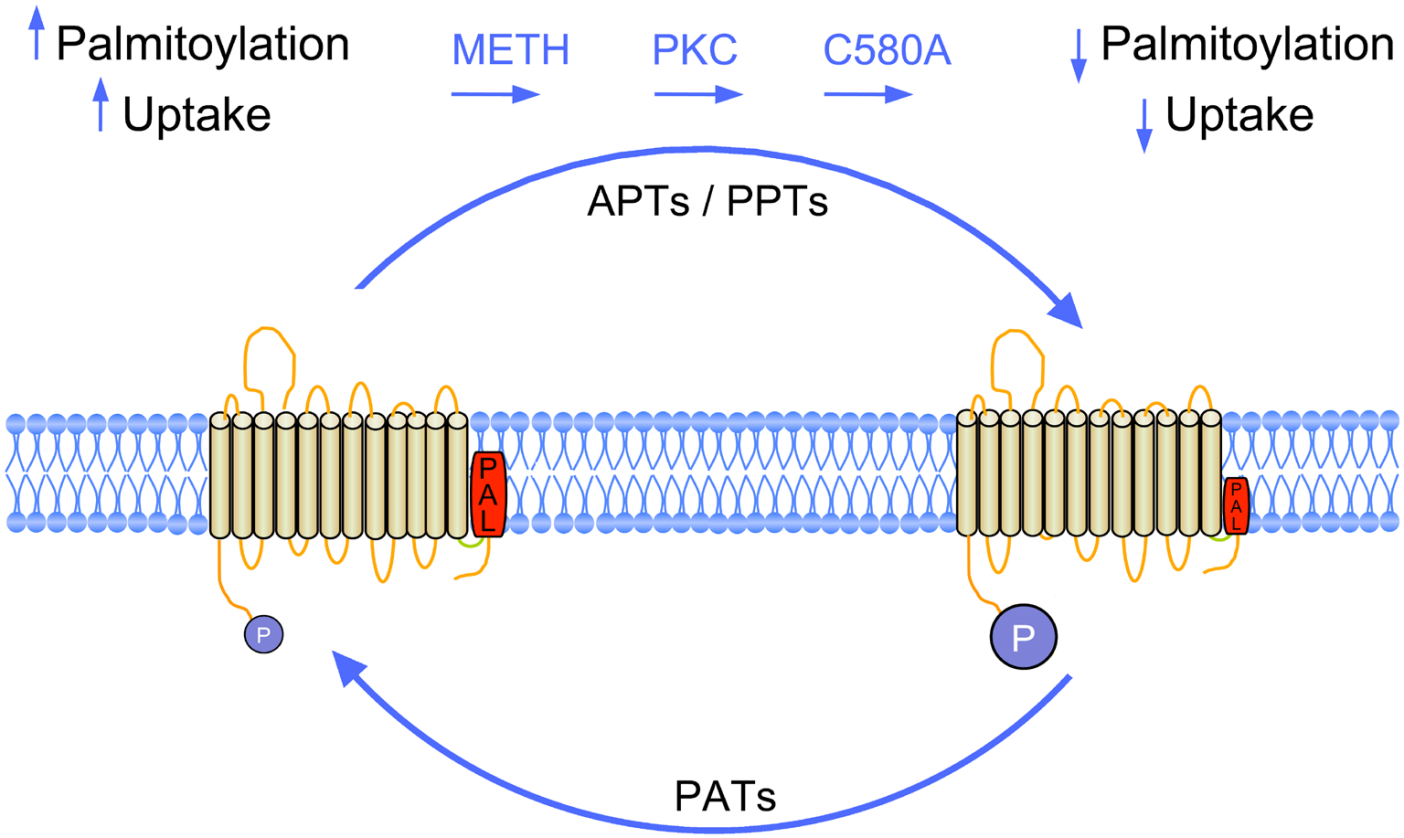
Model of DAT regulation by palmitoylation and METH. DAT exists in populations possessing lower or higher levels of palmitoylation (red rectangles) driven by the actions of PAT and APT/PPT enzymes, with phosphorylation (blue circles) undergoing reciprocal responses as indicated. In the absence of exogenous stimulation (left) the tonic level of DAT palmitoylation is high and phosphorylation is low, promoting a form that possesses higher transport *V*_max_. In the presence of METH, activation of PKC, or mutation of Cys580, palmitoylation is reduced, phosphorylation is increased, and transport *V*_max_ is reduced (right).

How METH exposure results in reduction of DAT palmitoylation is not known. Protein palmitoylation is catalyzed by palmitoyl acyltransferases (PATs) and depalmitoylation is catalyzed by acyl protein thioesterases (APTs) and protein palmitoyl thioesterases (PPTs) [60, 61], such that the reduction of DAT palmitoylation could follow from reduced activity of PATs and/or enhanced activity of APTs or PPTs (Figure 10). These enzymes are themselves under posttranslational control [60], and although there is no current information relating METH to the activities of these enzymes, acute dysregulation of their actions could potentially follow from impacts on PKC or other regulatory enzymes through METH‐induced alterations in cytoplasmic Ca^2+^ [62, 63]. To date, five PATs that enhance DAT palmitoylation have been identified [14], suggesting these proteins as potential loci for involvement in these events. In addition to events driven by changes in enzyme activity, a non‐exclusive possibility is that drug‐induced acceleration of transport could alter palmitoylation levels by impacting modification site accessibility or regulome constituents.

This study has described a rapid dysregulatory DAT palmitoylation response following an acute exogenous stimulus, but the findings support the potential for involvement of transporter palmitoylation in additional conditions such as long‐term drug use, endogenous reuptake disorders, or regional/sex‐specific transporter regulation. DAT displays pleiotropic responses to palmitoylation, with acute alterations linked to rapid transport regulation as described in this study, whereas chronic genetic or pharmacological suppression leads to enhancement of transporter degradation and steady‐state reductions in total levels [13, 14]. As such, the transient suppression of DAT palmitoylation by acute METH suggests the potential for chronic METH to induce long‐term reductions in palmitoylation that could lead to the reduced levels of transporter expression and reuptake capacity that are hallmarks of METH abuse [64, 65].

Palmitoylation could potentially also be impacted by endogenous conditions that establish differential reuptake or regulatory baselines. Many PAT, APT, and PPT enzymes are subject to mutations and expression deficits that result in improper modification and function of targets, with neuronal substrates including synaptic constituents such as receptors, channels, SNAREs, and scaffolds. Dysregulated palmitoylation of these proteins has been associated with diseases including schizophrenia, attention deficit hyperactivity disorder, and major depressive disorder [66, 67], supporting that similar impacts on DAT could contribute to imbalanced reuptake in DA disorders. Dysregulated DAT palmitoylation could also represent a mechanism underlying anomalous kinetics that have been observed in DAT polymorphic variants associated with DA imbalance conditions. In this regard, we have found that hDAT isoform A559V identified from individuals diagnosed with attention deficit hyperactivity disorder and bipolar disorder [68, 69] displays tonically reduced in vitro palmitoylation (unpublished result) that could relate to its altered kinetic properties, AMPH insensitivity, and enhanced phosphorylation. Increasing evidence also supports differential tonic regulation of DAT between brain regions and sexes that impacts neurochemical and behavioral outcomes [70, 71, 72]. In some cases, these differences have been linked to dysregulated transporter phosphorylation, which, by extension, supports potential involvement of palmitoylation.

The modification of DAT by palmitoylation thus represents a key component of the transporter regulatory machinery that could function in spatial and temporal control of DA clearance in normal and pathophysiological states. Further elucidation of palmitoylation mechanisms in DAT kinetic control and membrane trafficking, relationship to psychostimulant drugs, and responses to genetic conditions will clarify its broader physiological role and its potential as a therapeutic target in dopamine disorders.

## Author Contributions

R.A.V. and J.D.F. conceived experiments, analyzed data, and wrote the paper. M.J.H., D.E.B., C.D.K., M.S., A.C.B., M.Y.G., S.A.G., and C.R.B. performed experiments, analyzed data, and assisted in manuscript writing.

## Disclosure

The authors have nothing to report.

## Ethics Statement

All procedures involving animals were approved by the University of North Dakota Institutional Animal Care and Use Committee in accordance with the National Institutes of Health Guide for the Care and Use of Laboratory Animals. Experiments involving radioactivity were approved by the University of North Dakota Radiation Safety Committee, and experiments involving recombinant transporters were approved by the University of North Dakota Institutional Biosafety Committee.

## Conflicts of Interest

The authors declare no conflicts of interest.

## Data Availability

The data that support the findings of this study are included in the Materials, Methods, and Results sections of this article.
